# Gauging the influence of increased search effort on reporting rates of bottlenose dolphin (*Tursiops truncatus*) strandings following the deepwater horizon oil spill

**DOI:** 10.1371/journal.pone.0199214

**Published:** 2018-06-21

**Authors:** Jonathan L. Pitchford, Michael Garcia, Eric E. Pulis, Ashley Millan Ambert, Andrew J. Heaton, Moby Solangi

**Affiliations:** 1 Grand Bay National Estuarine Research Reserve, Moss Point, Mississippi, United States of America; 2 The Institute for Marine Mammal Studies, Gulfport, Mississippi, United States of America; 3 NOVACES LLC, New Orleans, Louisiana, United States of America; 4 University of South Alabama, Mobile, Alabama, United States of America; University of Missouri Columbia, UNITED STATES

## Abstract

The co-occurrence of the Deepwater Horizon oil spill and the northern Gulf of Mexico cetacean Unusual Mortality Event have raised questions about the stability of inshore bottlenose dolphin (*Tursiops truncatus*) populations throughout the region. Several factors could have contributed to the ongoing event, but little attention has been paid to the potential effects of increased search effort and reporting of strandings associated with oil spill response activities, which were widespread for an extended period. This study quantified the influence of increased search effort by estimating the number of bottlenose dolphin strandings reported by oil spill responders and comparing monthly stranding rates with and without response-related records. Results showed that response teams reported an estimated 58% of strandings during the Active Response period within the study area. Comparison of Poisson rates tests showed that when responder-influenced stranding records were removed, the monthly stranding rates from the Active Response period (May 2010 –April 2014) were similar to the Post-Removal Actions Deemed Complete period (May 2013 –March 2015) (e.g., *p* = 0.83 for remote areas in Louisiana). Further, analyses using the Getis-Ord Gi* spatial statistic showed that when response-related stranding reports were removed from the Active Response period, significant spatial clustering of strandings (*p* < 0.05) was reduced by 48% in coastal Louisiana. Collectively, these results suggest that increased search effort resulting from the Deepwater Horizon oil spill response throughout remote portions of the Unusual Mortality Event geographic region had the capacity to increase reporting and recovery of marine mammal strandings to unusually high levels. To better understand how stranding data relates to actual mortality, more work is needed to quantify dolphin population size, population trends, and carcass detection rates including the role of search effort. This is vital for understanding the status of a protected species within the northern Gulf of Mexico.

## Introduction

The longest running significant mortality event (also known as unusual mortality event (UME)) of marine mammals on record in the northern Gulf of Mexico (nGOM) occurred from March 1, 2010 (start date declared in December 2010) to July 31, 2014 (ending date declared in April 2016) and included 1,141 cetacean strandings over a region extending from the western border of Louisiana to Franklin County, FL [1]. This UME was the longest on record and the geographic extent was larger than the majority of declared UMEs [2]. Several factors have been suggested as contributors to this UME including, but not limited to a prolonged bout of cold weather in 2010 combined with unusually large freshwater floods in 2011 [3] and the Deepwater Horizon (DWH) oil spill that began on April 20, 2010 [4 – 6]. During the nGOM UME, cetacean strandings were documented in the UME geographic region with 614 occurring in Louisiana (LA), 318 in Mississippi (MS), 169 in Alabama (AL), and 40 on the Florida (FL) panhandle (bottlenose dolphins are not considered part of the UME in FL). The criteria used to define this UME was the unusual magnitude of strandings, which exceeded average yearly stranding rates from 2002–2009 plus 2 standard deviations [2]. An increase in strandings of this magnitude suggests that mortality rates of cetaceans increased; however, deriving accurate mortality rates from stranding data is difficult as the number of carcasses documented often represents a small fraction of total mortality [7–11]. Underreporting stems from several factors that can influence the detectability and documentation of carcasses including, but not limited to scavenging, drift, remoteness of the area, visibility [7–11], burying or removal of carcass [8], and carcass size [12]. Although the role of increased search effort has also been acknowledged as a potential factor that could increase carcass recovery rates [10,13], this has not been formally studied in the Gulf of Mexico since the DWH oil spill.

Following the DWH oil spill, Shoreline Cleanup Assessment Technique (SCAT) teams provided an internationally recognized oil spill response throughout the Deepwater Horizon Area of Response (AOR) [14]. This response included surveys of the shoreline from the Texas-Louisiana border in the west, to the Florida panhandle in Wakulla County in the east, which included approximately 7,049 km of shoreline. In the first few months of the spill response, nearly 48,000 people were participating in the response across the AOR. The number of responders gradually decreased to 6,600 by December 2010, and was continuously reduced as response activities concluded [15]. By July 2011, there were approximately 1,000 responders combing the beaches and that number held steady until early 2013 as response activities concluded in MS, AL, and FL [16]. On June 10, 2013, all removal actions were deemed completed by the U.S. Coast Guard (USCG) Federal On-Scene Coordinator (FOSC) for MS, AL, and FL, and on April 15, 2015 removal actions were deemed complete in Louisiana.

Although approximately 7,049 km of shoreline across the AOR were surveyed by SCAT, much of it was found to have not been affected by the spill. SCAT found that approximately 5,283 km were never oiled per the DWH SCAT database [17]. Shoreline Treatment Recommendations (STR) were developed for shorelines that required some level of clean-up. This resulted in 1,252 km of shoreline receiving SCAT surveys and Operations until the removal actions were deemed complete by the DWH FOSC. The oil spill response included a variety of activities including but not limited to: skimming and booming operations in 2010, beach clean-up after oil became stranded on the shore in 2010, beach patrol and maintenance to clean residual oiling along the shore from 2011–2014, aerial surveillance, and response-related projects such as shoreline investigation and oil removal projects. Barring severe weather and FOSC directed stand-downs, response teams conducted daily operations within the AOR from April 20, 2010 –April 15, 2014 [16–17]. Response teams varied in size and coverage area based on the response activity. These teams, ranging in size from four to more than 20 responders, conducted shore-based surveys along the entire length of a SCAT segment including the supratidal, intertidal, and subtidal zones along the shoreline. In addition, there were other response teams conducting water response activities and aerial surveillance across the AOR.

The extent and duration of the oil spill response was extensive and included regular surveys and response activities throughout the region from beaches accessible to the public by car to isolated salt marshes and barrier islands accessible only by boat. Venn-Watson et al. [5] identified clusters of cetacean strandings indicative of large-scale mortality in northern coastal LA and MS from March–May 2010, Barataria Bay, LA from August 2010 –December 2011, and in coastal MS and AL from January–December 2011. Areas in Barataria Bay, MS, and AL received heavy oiling during these periods and associated response efforts were heightened in these locations due to the presence of oil [14]. The LA, MS, and AL coasts include remote regions not often accessed by the public (e.g., isolated salt marsh channels, barrier islands) and stranding reports from these areas has historically been sparse [1]. Oil spill responders were directed to report injured and stranded wildlife found at or near their investigation and clean-up sites. As such, it is reasonable to assume that the DWH oil spill response would increase carcass detection and reporting of stranded cetaceans; however, no study (that we are aware of) has attempted to quantify the influence of responders on reporting rates.

While the potential effect of response activities on the number of reported strandings following the DWH oil spill has been discussed in the literature, no attempt has been made to quantify the influence of response activities and subsequent increased search effort on carcass detection. A “coverage factor” was used by Venn-Watson et al. [5] in regression models to account for different levels of coverage within the Southeastern Marine Mammal Stranding Network (i.e., 0 = no coverage, 1 = limited coverage, and 2 = full coverage). This approach was used primarily to account for limited stranding response in areas without stranding facilities or suffering from hurricane impacts [5], but was not used to account for varying levels of search effort (i.e., number of people searching, time spent searching, etc.) following the oil spill. There is limited information available regarding stranding search effort prior to the onset of the DWH response, which makes quantifying detection rates difficult.

The objective of this study was to examine the influence of responders and associated search effort on reporting of stranded bottlenose dolphins within the AOR following the DWH oil spill. This was accomplished by 1) estimating the number of strandings located and/or reported by oil spill responders using Level A data comments and detailed records of response activities (i.e., location of response teams on specific dates), 2) graphically and statistically analyzing the potential effects of responders on monthly stranding rates, and 3) analyzing the potential influence of responders on the spatial clustering of strandings. Accomplishing these objectives should help to clarify the role of increased observer effort following the DWH oil spill, which is vital for improving estimates of carcass recovery and mortality rates of protected bottlenose dolphins in the nGOM.

## Materials and methods

### Study area

**Fig 1:**
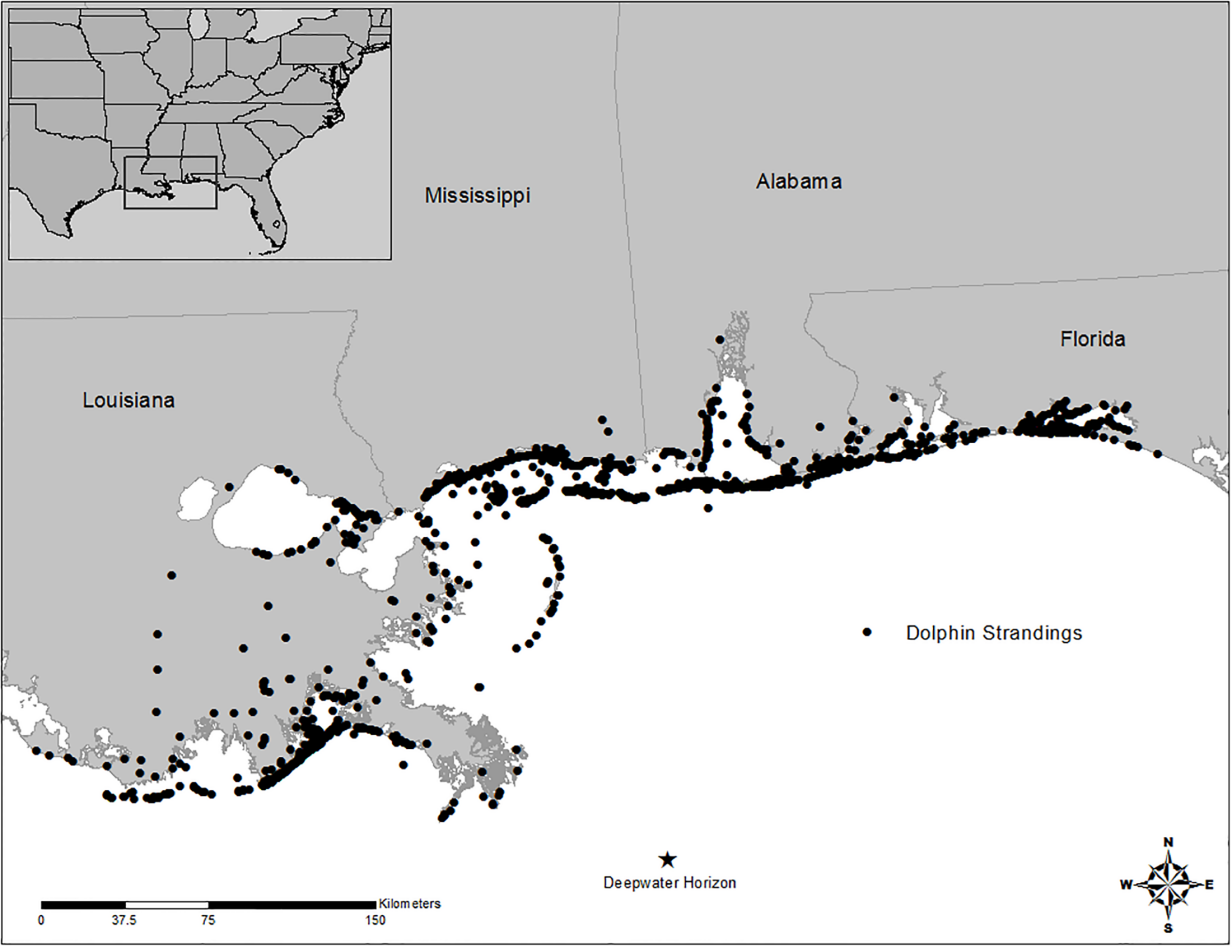
Location of dolphin strandings from 1996–2015 that were used to analyze the influence of increased search effort associated with the Deepwater Horizon oil spill response in the northern Gulf of Mexico.

The study area for this project aligns with the primary AOR delineated in Michel et al. [14] and includes approximately 7,049 km of shoreline that was surveyed and monitored heavily by SCAT and Operations teams (Fig 1). This area was also within the geographic boundaries of the nGOM UME that was declared by the National Oceanic and Atmospheric Administration (NOAA) National Marine Fisheries Service (NMFS) that began in March, 2010 [5].

### Level A data acquisition

Level A data (copy of form available at http://www.fisheries.noaa.gov/gpea_forms/pr/0178_mmstranding_rpt.pdf) were obtained from the Marine Mammal Health and Stranding Response Program (MMHSRP) [1], which is a repository for data received from state Marine Mammal Stranding Networks (MMSN). These are basic records for each stranded marine mammal that include geographic coordinates, sex of the animal, body condition (i.e., decomposition code), body length, and in some cases includes information about the responding party. For this study, complete Level A stranding data from 1996–2015 were obtained for LA, MS, AL, and FL and were filtered to exclude areas outside the AOR. The final data set included strandings from LA (Terrebonne, Lafourche, Plaquemines, St. Bernard, Orleans, and St. Tammany parishes), MS (Hancock, Harrison, and Jackson counties), AL (Mobile and Baldwin counties), and FL (Escambia, Santa Rosa, Okaloosa, and Walton counties). Level A data records used in further analyses included the date the stranding was reported, geographic coordinates, and data comments.

### Data filtering

A total of 7,263 marine mammal stranding records were obtained from the MMHSRP website from 1996–2015. Level A data contained a wide variety of formats for geographic coordinates (e.g., degrees minutes seconds, degrees decimal minutes, etc.). As such, an initial step included conversion of all coordinates to decimal degrees. Coordinate accuracy was vital for this project, thus data were first filtered to remove all records with missing or erroneous coordinates resulting in a total of 7,111 stranding records. Further, live strandings and all strandings other than bottlenose dolphins were filtered from the dataset resulting in a total of 5,635 stranding records. Remaining stranding coordinates were imported into ESRI ArcMap 10.3^™^ and all plotted strandings from LA, MS, AL, and FL that fell outside of the AOR were removed, which resulted in a total of 1,996 stranding records. Finally, records that plotted in locations that did not match descriptive information (e.g., stranding plotted offshore, but descriptive information indicated that the stranding occurred on a specific beach) were removed resulting in a total of 1,941 stranding records that were used in the following analyses (S1 Table).

### Linking stranding and responder data

To analyze the influence of oil spill responder surveillance on reported stranding observations, three temporal groups that corresponded to the DWH oil spill were selected. These groups were: Pre-Spill, Active Response, and Post-Removal Actions Deemed Complete (RADC). The Pre-Spill group contained stranding records for the period prior to the DWH oil spill, from January 1996 to April 2010. The Active Response group contained records during active clean-up and the Post-RADC group included those that were reported after clean-up was deemed complete. The Active Response and Post-RADC groups contained stranding records that occurred from May 2010 –April 2015 (Table 1). The transition from Active Response to Post-RADC varied across the AOR as areas were declared complete (i.e., RADC) by the USCG FOSC.

**Table 1:** Distribution of stranding records among geographic subgroups during the Active Response and Post-Removal Actions Deemed Complete (Post-RADC) time periods as it relates to the oil spill response.

Geographic Subgroup	Pre-Spill^1^	Active Response Dates (mm/yyyy)	Active Response	Post-RADC Dates (mm/yyyy)	Post-RADC^2^	Total
LA-Public/Private Beaches	123	05/2010–03/2014	149	04/2014–04/2015	22	294
LA-Remote Areas	109	05/2010–12/2013	185	01/2014–04/2015	29	323
MS-Barrier Islands	109	05/2010–04/2013	75	05/2013–04/2015	11	195
AL-Mobile County	82	05/2010–05/2013	56	06/2013–04/2015	14	152
AL-BSNWR^3^ to Orange Beach	51	05/2010–05/2013	23	06/2013–04/2015	21	95
Total	474	-	488	-	97	1059

^1^The Pre-Spill period used in this study was 1/1/1996–4/30/2010

^2^Removal Actions Deemed Complete

^3^Bon Secour National Wildlife Refuge

**Fig 2:**
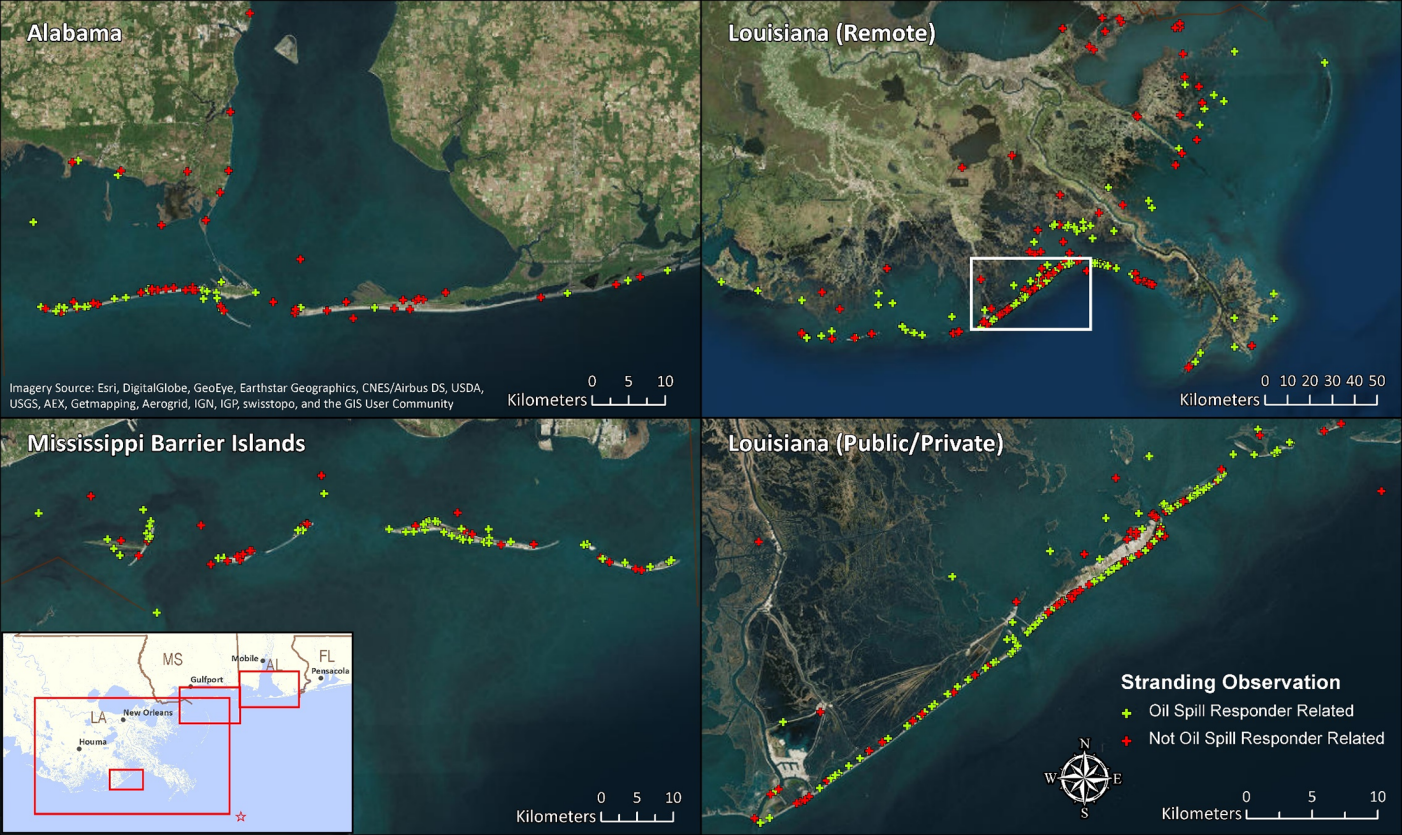
Stranding observations in Alabama, Mobile County and Baldwin County—Bon Secour to Orange Beach, Louisiana (remote), Mississippi Barrier Islands, and Louisiana (public/private) from 2010 –April 2015. Green points are oil spill response-related observations and red points either did not specify the reporting party or specifically noted that they were not related to the oil spill response effort.

Five geographic subgroups were selected that correspond with DWH areas of operations. These were: LA–Public/Private Beaches, LA–Remote Areas, MS–Barrier Islands, AL–Mobile County, and AL–Bon Secour National Wildlife Refuge (BSNWR) to Orange Beach, AL (Fig 2). A subgroup was not selected for FL because bottlenose dolphins were not part of the nGOM UME in FL and initial analyses of Level A data confirmed that the monthly stranding rates in FL did not increase during the study period.

The Level A data were examined for stranding records during the Active Response period to identify records that were reported by oil spill responders. This was done by reviewing the Level A data comments to determine if an oil spill responder played a role in the reporting of a stranding. Specifically, the Level A data comments within the Active Response group were examined to determine if the records noted that the reporting party was related to the oil spill, not related to the oil spill, or if the relation was not specified. Examples of comments that indicated that response teams located or reported a carcass included: “initially discovered by SCAT team…”, “carcass was found … by Task Force 3.”, “Carcass was reported by BP work crew,” etc. Conversely, some comments specifically indicated that the reporting party was not affiliated with the response. Those records were marked as not response related. Stranding records that did not have a reporting party specified in the Level A data comments were further reviewed using the DWH SCAT database, Operation’s database, and Incident Action Plans [16–17]. Stranding observations that were within a 161 m (or 1/10^th^ of a mile) radius of oil spill response workers on the day the stranding was reported were noted as being response-related. A 161 m radius was selected because responders within this distance would likely have detected and reported strandings as they patrolled large areas beyond this radius while surveying SCAT segments. Finally, strandings noted in the Level A data comment review and the oil spill response database review were compiled to identify all stranding records during the Active Response period that were reported by oil spill responders (hereafter referred to as “response-related”) (S2 Table).

### Analyses of monthly stranding rates

The total number of strandings during the Active Response period was compared to the Post-RADC period within each geographic subgroup. Strandings during the Active Response contained responder-influenced observations and the Post-RADC period did not. This comparison was used to determine if responders had a significant impact on the number of observations recorded during the Active Response period compared to the Post-RADC period. Strandings from the Active Response period were also compared with Pre-Spill strandings to understand the influence of responders. Pre-Spill strandings contained records from 1996 to 2010, which included prior UME strandings and several periods of assumed low observation activity. As such, a five-year period of Pre-Spill strandings that were not affected by UMEs (May 2005 –April 2010) were compared with strandings from the Active Response period.

Stranding data are count data that can be described using the Poisson distribution. As such, the Pearson Chi-Square goodness-of-fit test was used to determine a good fit to the Poisson distribution [18] prior to conducting statistical analyses. This was done using a “Goodness-of-Fit Test for Poisson” provided in Minitab^®^ 17.2.1.

The responder-influenced analysis was performed using a graphical and analytical approach. The graphical approach consisted of plotting the data using a U-chart and staging the mean and control limits along the temporal groupings of the selected geographic subgroups. That is, each temporal group was displayed with a mean monthly stranding rate and control limits calculated independently of the other groups. Displaying the data in this way provided an informative view of the variation within each group. The U-Chart tool provided in Minitab^®^ 17.2.1 was used for graphical display.

Stranding rates were compared statistically using the “Comparison of Poisson rates” test within the program R 3.2.1. The R implementation of this test was selected because it provided confidence intervals and an alternative hypothesis that the true rate ratio was different than 1. The R function *poisson*.*test* was used to conduct a two-tailed test such that H_0_: λ_1_ = λ_2_ where λ is the rate of occurrence of monthly stranding observations within a temporal grouping. A 95% confidence interval was calculated in the analysis. Each of the five geographic subgroups was tested such that they are a family of hypotheses. Testing these five hypotheses separately increases the risk of making a type 1 error. To correct for this, the Bonferroni correction was used to adjust the alpha risk such that α’ = α/5 = 0.05 / 5 = 0.01.

### Spatial analyses

Stranding records were plotted using a projected coordinate system (UTM Zone 15 N) for the Pre-Spill (January 1996 –April 2010; n = 1,065), Active Response (May 2010 –April 2014; n = 695), Active Response excluding response-related records (May 2010 –March 2014; n = 367), and Post-RADC (May 2013 –March 2015; n = 199) time periods as described above. To quantify the spatial clustering of strandings, we used Incremental Spatial Autocorrelation (ISA) and the Getis-Ord Gi* spatial statistic within the program ESRI ArcMap 10.3^™^ [19–20]. This was done by first aggregating stranding locations within a 25 km radius across the AOR using the ‘Integrate’ and ‘Collect Events’ tools. Aggregated stranding records were used in ISA to help identify the smallest distance where spatial clustering was most pronounced for each temporal grouping. This distance was used as the threshold distance in the Getis-Ord Gi* statistic, which works to identify autocorrelation within a pre-defined neighborhood (e.g., peak distance determined in ISA) and tests a hypothesis that no spatial clustering is occurring over the extent of the input features. The output consists of a Z-score and P-value for each input feature, where large positive Z-scores (> 1.96) represent spatially significant hotspots (*p* < 0.05) and small negative Z-scores (< -1.96) represent spatially significant cold spots (*p* < 0.05) [21]. The equation for Getis-Ord Gi* is:

$$G_{i}^{*}\;=\;\frac{\sum_{j\;=\;1}^{n}w_{i,j\;}x_{j}-\;\overset{-}{X}\sum_{j\;=\;1}^{n}w_{i.j}}{S\sqrt{\frac{n\;\sum_{j\;=\;1}^{n}w_{i,j}^{2}-(\sum_{j\;=\;1}^{n}{w_{i,j})}^{2}}{n-1}}}$$ (1)

where *x*_*j*_ is the value for each feature, *w*_*i*,*j*_ is the spatial weight between features, n is the total number of features, and $\overset{-}{X}$ is the average value of the input features. The value for *S* is given by:

$$S\;=\;\sqrt{\frac{\sum_{j\;=\;1}^{n}x_{j}^{2}}{n}-}({\overset{-}{X})}^{2}$$ (2)

‘Euclidean distance’ was used as the distance method and ‘Zone of Indifference’ was used as the conceptualization of spatial relationships for Getis-Ord Gi* runs for each temporal grouping. To analyze changes in clustering across selected temporal groups, Z-scores were interpolated using the Inverse Distance Weighted (IDW) interpolation tool. A buffer, equal to the peak distance determined in ISA for each respective temporal group, was generated for integrated points and was used to clip the raster dataset for each temporal group.

Spatial analyses were run for all stranding records within each temporal group and a follow-up analysis was conducted for records in Louisiana classified as public/private and remote during the Active Response period. The purpose of the follow-up analysis was to compare spatial clustering of strandings during the Active Response period both with and without response-related records.

## Results

### Effects of increased surveillance on stranding observations

There were 488 total stranding observations during the Active Response period in the geographic groups analyzed (Table 2). Of these, the Level A data comments were used to identify 159 records (33%) as having been reported by oil spill responders. There were 48 records (10%) in the Level A data that specifically noted that the reporting party was not related to the oil spill response. The remaining records were cross-referenced to DWH response databases to identify occurrences where oil spill responders were within 161 m of the stranding observation on the day the stranding was reported, resulting in 122 records (24%) identified. The remaining 159 records (33%) could not be linked to oil spill response activities. In total, 281 of the original 488 stranding observations (58%) were determined to be response-related during the Active Response period. The spatial distribution of stranding records among selected geographic subgroups is shown is Fig 2.

**Table 2:** Distribution of stranding observations among observer parties during the Active Response period in the northern Gulf of Mexico 2010–2014.

Active Response—Observing Party	2010	2011	2012	2013	2014	Total
Not response-related^1^	1	22	8	15	2	48
Response-related^2^	27	79	22	25	6	159
161 m responder^3^	24	59	23	16	0	122
Unknown relation^4^	22	39	41	47	10	159
Total	74	199	94	103	18	488

^1^ Not response-related—Level A data specified a reporting party that was not related to the oil spill

^2^ Response-related—Level A data specified a reporting party that was related to the oil spill

^3^ 161 m responder—Oil spill responders where within 161 m of stranding observation, when Level A data did not specify the reporting party

^4^ Unknown relation–Reporting party was not specified and responders were not located within 161 m based on the DWH Incident Action Plans, or Operations and SCAT databases.

**Fig 3:**
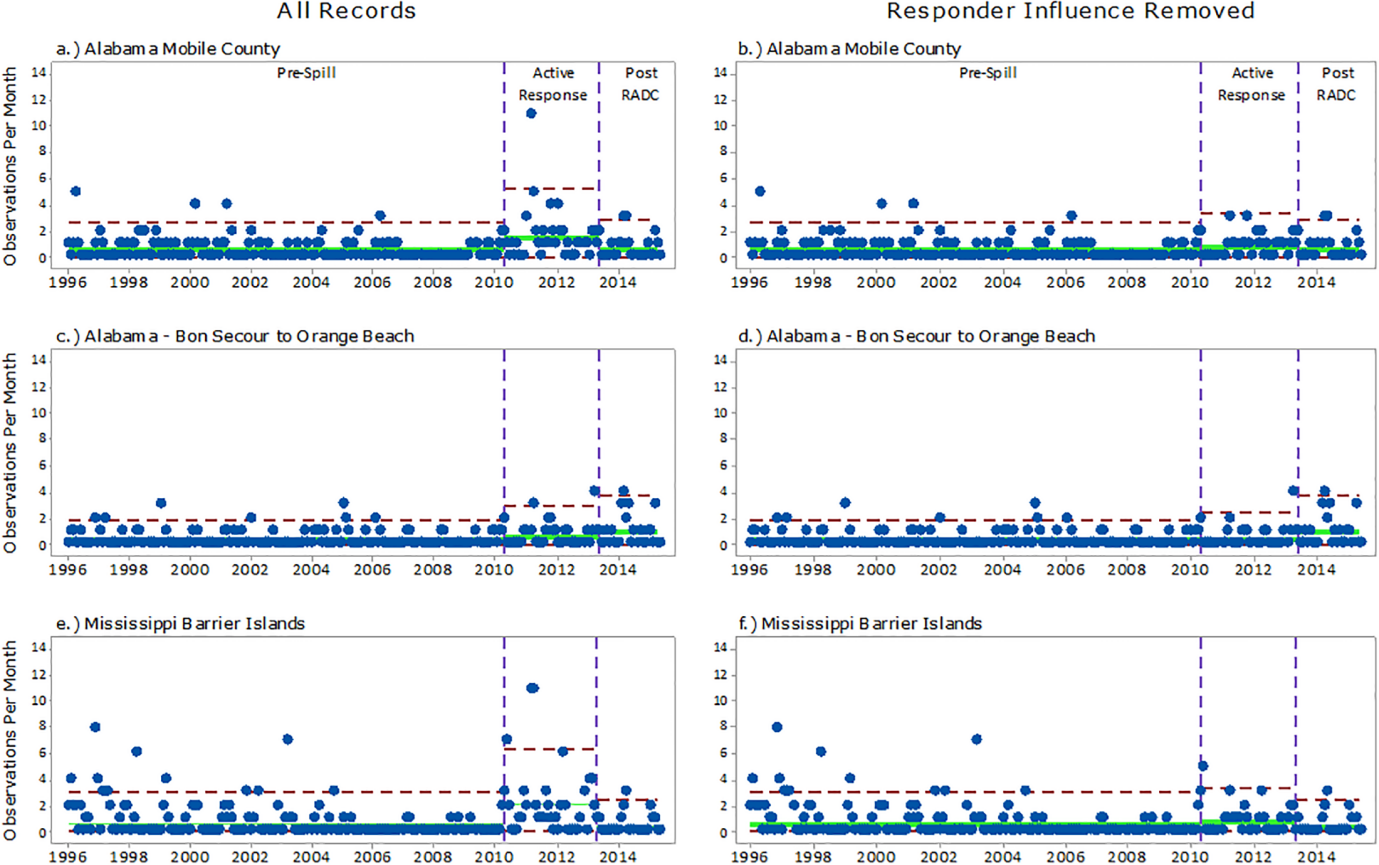
U-Charts displaying the temporal distribution of strandings in the northern Gulf of Mexico from Jan 1996 –Apr 2015 for a) Alabama Mobile County with all records, b) Alabama Mobile County with response-influenced records removed from the Active Response period, c) Alabama—Bon Secour to Orange Beach with all records, d) Alabama—Bon Secour to Orange Beach with response-influenced records removed from the Active Response period, e) Mississippi Barrier Islands with all records, and f) Mississippi Barrier Islands with response-influenced records removed from the Active Response period. Green lines represent the mean monthly stranding rate and red lines are three standard deviations above the mean.

**Fig 4:**
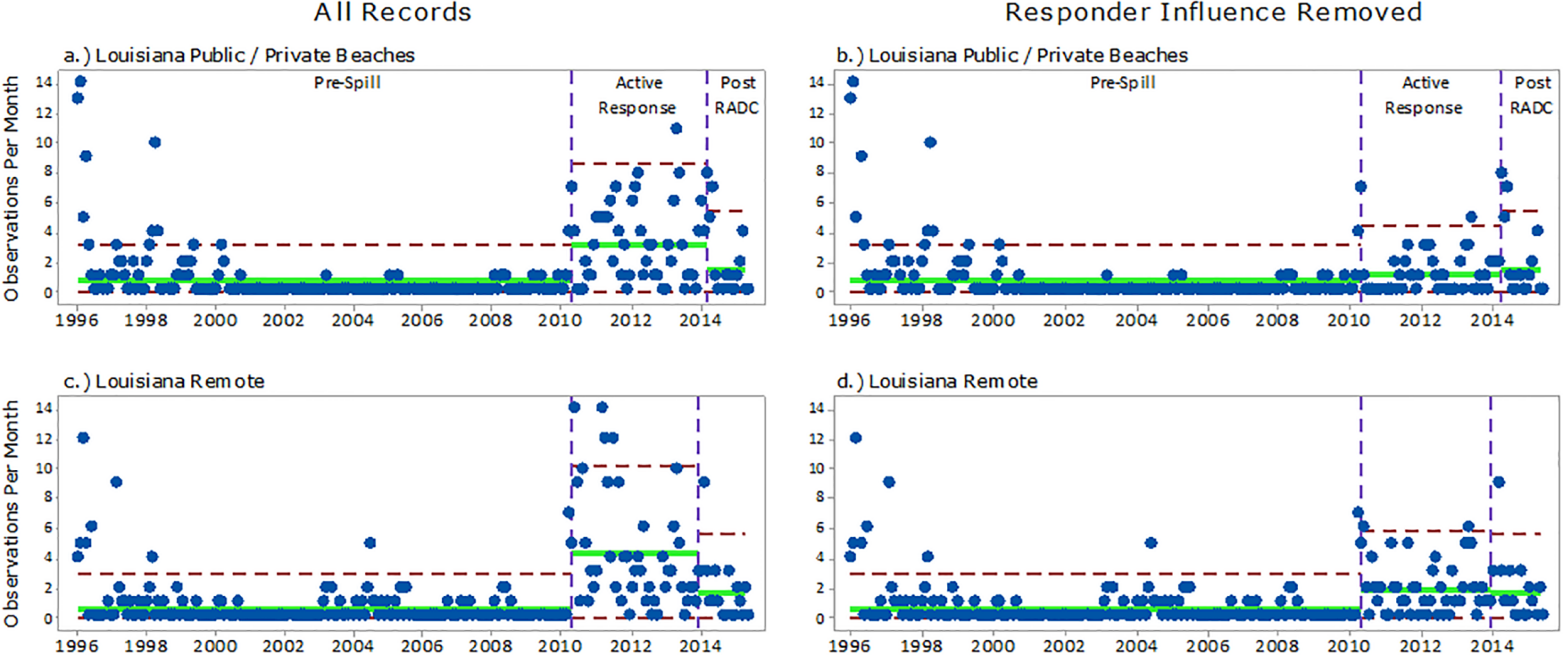
U-Charts displaying the temporal distribution of strandings in the northern Gulf of Mexico from Jan 1996 –Apr 2015 for a) Louisiana Public/Private Beaches with all records, b) Louisiana Public/Private Beaches with response-influenced records removed from the Active Response period, c) Louisiana Remote areas with all records, and d) Louisiana Remote areas with response-influenced records removed from the Active Response period. Green lines represent the mean monthly stranding rate and red lines are three standard deviations above the mean.

Figs 3 and 4 (left column) provide a graphical view of the rate of monthly stranding observations increasing from the Pre-Spill period to the Active Response period. Monthly stranding rates increased at Bon Secour to Orange Beach, Mobile County, MS Barrier Islands, Louisiana Public/Private, and Louisiana Remote by factors of 2.07, 3.15, 3.30, 4.40, and 6.67, respectively (Table 3). In contrast, a sharp decrease in monthly observation rates was seen from Active Response period to Post-RADC across the geographic subgroups, with the exception of AL Bon Secour to Orange Beach.

**Table 3:** Monthly stranding rates (strandings per month) among geographic subgroups during the Pre-Spill, Active Response (all data), Active Response with responder-influenced records removed, and Post-Removal Actions Deemed Complete (RADC) periods in the northern Gulf of Mexico from Jan 1996 –April 2015.

Geographic Subgroup	Pre-Spill	Active Response- all data^1^	Active Response—responder influenced records removed^2^	Post-RADC
AL—Bon Secour to Orange Beach	0.30	0.62	0.43	0.88
AL—Mobile County	0.48	1.51	0.76	0.58
MS—Barrier Islands	0.63	2.08	0.78	0.44
LA—Public/Private Beaches	0.72	3.17	1.17	1.57
LA—Remote Areas	0.63	4.20	1.82	1.71

^1^Monthly stranding rates calculated using all stranding records for selected areas

^2^Monthly stranding rates calculated after responder-influenced records were removed

The stranding records in the Active Response period that were identified as being response-related were removed and displayed on the right column of Figs 3 and 4. After removal of response-related records, the rate of monthly stranding observations for the Active Response period was similar to those in the Post-RADC period (Table 3). A ‘Comparison of Poisson rates’ test was used to compare this statistically. Prior to performing the Poisson rate comparison, a Pearson Chi-Square goodness-of-fit test was conducted on each geographic subgroup to determine if the data fit the Poisson distribution. The results indicated an approximate fit for each subgroup (Table 4).

**Table 4:** Results of a Pearson Chi-Square goodness-of-fit test for selected geographic subgroups during the Active Response and Post-Removal Actions Deemed Complete time periods.

Geographic subgroup	Active Response p-values	Post-RADC p-values
AL—Bon Secour to Orange Beach	0.658	0.183
AL—Mobile County	0.748	0.428
MS—Barrier Islands	0.406	0.126
LA—Public/Private Beaches	0.012	0.037
LA—Remote Areas	0.049	0.100

The ‘Comparison of Poisson rates’ test was performed on each geographic subgroup by comparing the Active Response and Post-RADC periods with a two-tailed test such that the null hypothesis H_0_: λ_1_ = λ_2_, where λ was the rate of monthly stranding observations within each group. The results showed that strandings during the Active Response period and the Post-RADC period were not significantly different when the response-related observations were removed in each geographic subgroup (Table 5).

**Table 5:** Results of a Comparison of Poisson Rates test for the Active Response (with response-related strandings removed) and Post-Removal Actions Deemed Complete (RADC) time periods. No *p* values were significant at the Bonferroni-corrected level of 0.01. Also shown are monthly stranding rates and associated variance for the Pre-Spill period (May 2005 –April 2010).

Comparison of Poisson rates Active Response = Post-RADC	p-value	95% LCI^1^	95% UCI^1^	Sample Rate Ratio^2^	Pre-Spill mean monthly stranding rate^3^	Variance^4^
AL—Bon Secour to Orange Beach	0.042	0.24	0.99	0.49	0.17	0.28
AL—Mobile County	0.528	0.66	2.67	1.30	0.37	0.44
MS—Barrier Islands	0.142	0.85	3.93	1.77	0.25	0.26
LA—Public/Private Beaches	0.277	0.45	1.28	0.74	0.32	1.14
LA—Remote Areas	0.831	0.69	1.69	1.07	0.42	1.43

^1^ 95% lower (LCI) and upper (UCI) confidence interval about the sample rate ratio

^2^ Sample rate ratio—Active Response monthly observation rate / Post-RADC monthly observation rate

^3^ Pre-Spill monthly stranding rate was calculated using stranding records for a five-year period before the oil spill (May 2005 –April 2010).

^4^ Variance calculated for the Pre-Spill monthly stranding rate

Strandings during the Active Response period were also compared to the Pre-Spill period using stranding records from five years prior to the spill (May 2005 –April 2010). Because the range of Pre-Spill data for this comparison was shorter than the range viewed in the U-Charts, the monthly stranding observation rates were re-calculated and the results are provided in Table 5. Prior to analyzing the Poisson rates of the Pre-Spill groups, the Pearson Chi-Square goodness-of-fit test was conducted. The MS Barrier Island (p = 0.06), AL Mobile County (p = 0.44), and AL Bon Secour to Orange Beach (p = 0.7) fit the Poisson distribution. The LA-Public/Private and LA-Remote subgroups (p < 0.001) did not fit the Poisson distribution and could not be analyzed; however, the Active Response stranding observation rates in LA were higher than the May 2005 –April 2010 Pre-Spill period, even with the response-related observations removed. The monthly stranding observation rate in LA Remote Areas increased by a factor of 4.3 from 0.42 per month to 1.82 per month. Likewise, stranding rates in LA Public/Private Areas increased by a factor of 3.7 from 0.32 per month to 1.17 per month.

Table 6 provides the results of Poisson rate tests for MS and AL. These results show that the Active Response stranding rates in the AL-Bon Secour to Orange Beach subgroup were similar to Pre-Spill stranding rates when response-related stranding observations were removed. However, Pre-Spill stranding rates for AL-Mobile County and the MS Barrier Islands were significantly different than the Active Response rate when response-related strandings were removed.

**Table 6:** Results of Comparison of Poisson Rates test used to compare stranding rates during the Active Response to the Pre-Spill time period for selected geographic groups.

Comparison of Poisson rates Active Response = Pre-Spill	Pre-Spill Stranding Rate	Active Response Stranding Rate	p-value	95% Lower Confidence Interval^1^	95% Upper Confidence Interval^1^	Sample Rate Ratio^2^
AL—Bon Secour to Orange Beach	0.17	0.43	0.140	0.80	3.76	1.73
AL—Mobile County	0.37	0.76	0.013	1.14	3.79	2.06
MS—Barrier Islands	0.25	0.77	<0.01	2.20	10.77	4.67

^1^ 95% confidence interval about the sample rate ratio

^2^ Sample rate ratio—Active Response monthly observation rate / Pre-Spill monthly observation rate

### Spatial analyses

**Fig 5:**
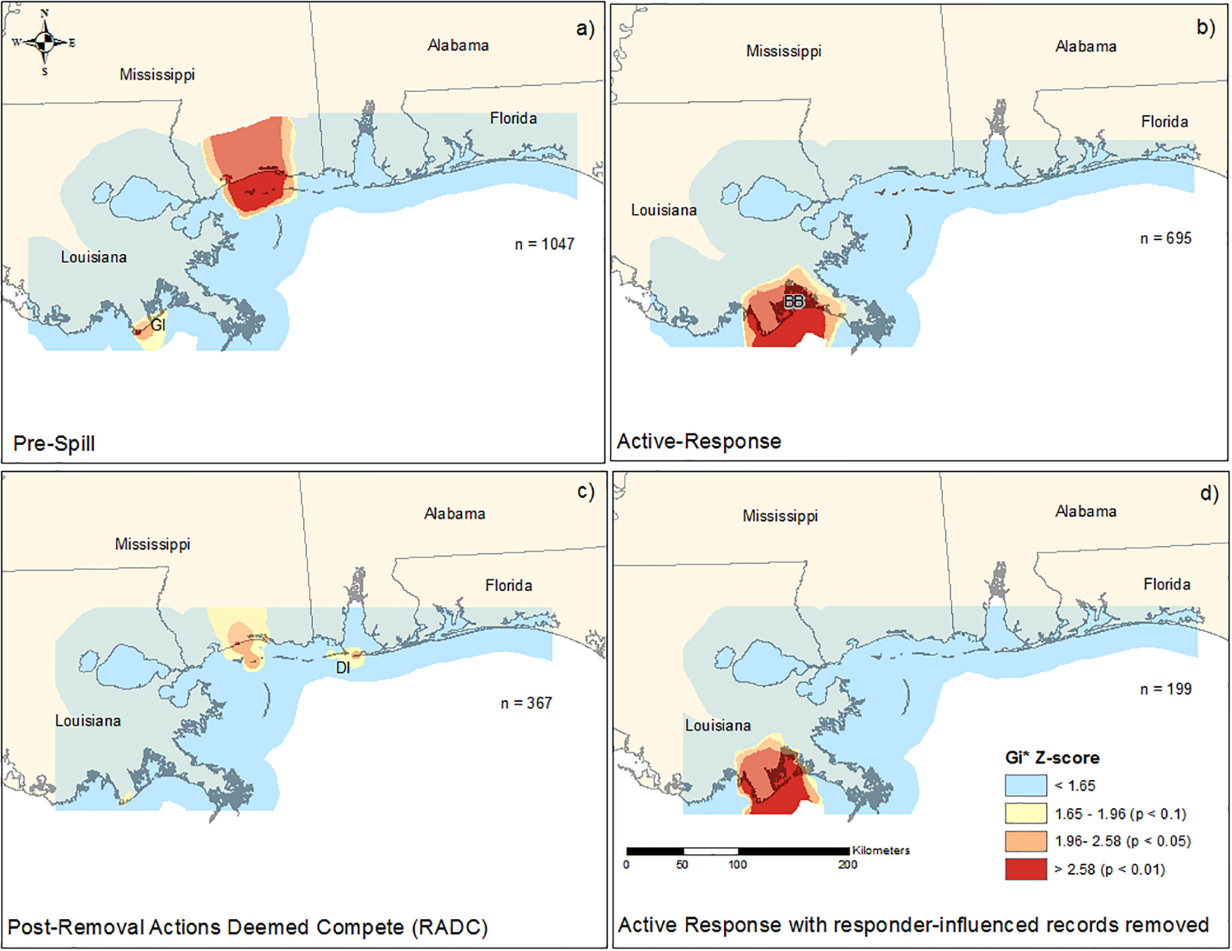
Maps showing the results of Getis-Ord Gi* statistical tests on stranding records for the a) Pre-Spill period from 1996 –April 2010, b) Active Response period from May 2010 –April 2014, c) post-Removal Actions Deemed Complete (Post-RADC) from May 2013 –March 2015, and d) Active Response period excluding records determined to be influenced by responders from May 2010 –March 2014. Each map shows the Gi* Z-score classified into four categories: non-significant clustering and significant clustering at the α < 0.1, 0.05, and 0.01 levels. Also shown is the sample size for each temporal grouping and labels for Grand Isle (GI), Barataria Bay (BB), and Dauphin Island (DI).

The results of ISA showed that spatial clustering of strandings was most pronounced at 30 km during the Pre-Spill period, 40 km during the Active Response, 30 km during the Active Response when excluding responder-influenced records, and at 30 km during the Post-RADC periods. Analyses of Pre-Spill stranding records using the Getis-Ord Gi* statistic helped to identify significant spatial clusters of strandings (*p* < 0.05) for the majority of the MS coastline including the barrier islands and in LA, primarily in the vicinity of Grand Isle, LA (Fig 5a). During the Active Response period, significant clustering was only detected in LA in a large area that includes Grand Isle, Barataria Bay, and portions of the MS River Delta in LA (Fig 5b). When excluding stranding records potentially influenced by response activities, the magnitude of the LA spatial cluster was reduced to Grand Isle and Barataria Bay (Fig 5c). Spatial clustering of strandings during the Post-RADC period was limited to approximately 20 km along the MS mainland coast and extending south to the barrier islands (Ship Island) and also included an area in the vicinity of Dauphin Island, AL (Fig 5d).

**Fig 6:**
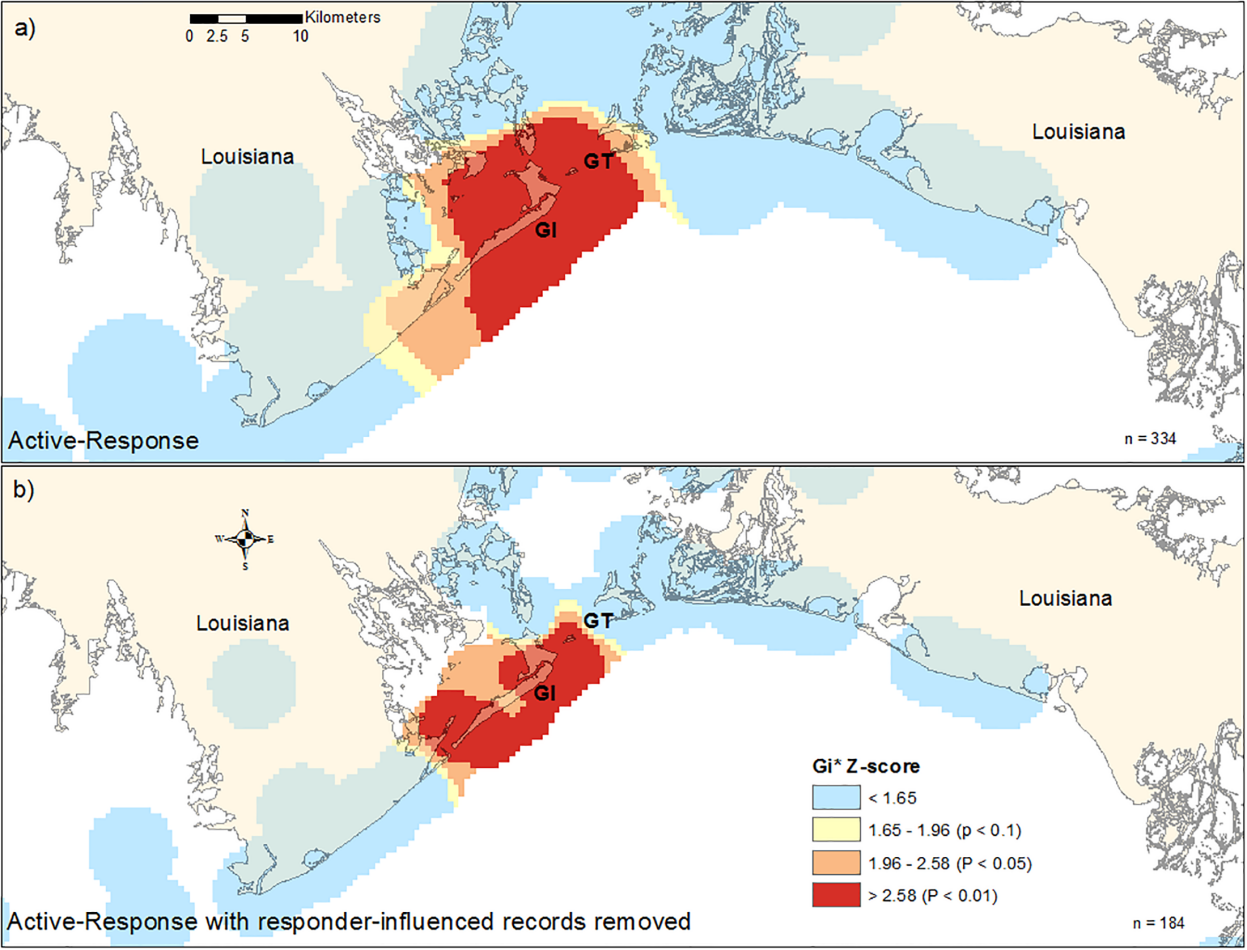
Maps showing the results of Getis-Ord Gi* statistical tests on stranding records in Louisiana for the a) Active Response period from May 2010 –April 2014 and b) Active Response period excluding records determined to be influenced by responders from May 2010 –March 2014. Each map shows the Gi* Z-score classified into four categories: non-significant clustering and significant clustering at the α < 0.1, 0.05, and 0.01 levels. Also shown is the sample size for each temporal grouping and labels for Grand Isle (GI) and the Grand Terre Islands (GT).

The results of ISA for the areas classified as LA public/private and LA remote showed that spatial clustering was most pronounced at 6 km during the Active Response and 4 km during the Active Response when excluding responder-influenced records. The results of Getis-Ord Gi* analyses showed significant spatial clustering (*p* < 0.05) in a 311 km^2^ area from Grand Isle, LA in the west to the Grand Terre Islands in the east, extending approximately 7 km into Barataria Bay to the north and 4 km into the nGOM to the south (Fig 6a). When excluding the responder-influenced records, significant spatial clustering was reduced 48% to 150.8 km^2^ (Fig 6b). The majority of clustering remained centered on Grand Isle, LA with reduced clustering to the east, north (Barataria Bay), and south (nGOM) when compared with clusters generated from all stranding records for this region.

## Discussion

Several studies have reported the occurrence of large-scale mortality of bottlenose dolphins in the nGOM after the DWH oil spill [2,5,6,22], but little is known about the influence of oil spill responders on the number of reported strandings. In this study, we estimated that 281 out of 488 strandings (58%) in the AOR during the Active Response period were located and/or reported by personnel involved in response-related activities. The summary of DWH response operations shows that large numbers of observers regularly accessed remote areas during the Active Response phase, which likely increased carcass detection rates. We found that when response-related observations were excluded, the monthly stranding rates during the Active Response phase were not significantly different than those after clean-up had been deemed complete. Higher stranding rates during the Active Response phase were likely related to response teams reporting strandings. Also, when response-related strandings were excluded, stranding rates before and during the Active Response were not significantly different in the Bon Secour to Orange Beach area. Given that stranding rates did not increase in FL from Pre-Spill through Active Response, these findings suggest that strandings may have only increased on the Mississippi Sound barrier islands (including Dauphin Island) and southeast Louisiana.

Spatial analyses of the Pre-Spill period identified a significant cluster of strandings along the MS coast and barrier islands and also in accessible areas along the LA coast near Grand Isle. These clusters could be a function of the size of the local dolphin populations in these areas [23–25] and prevailing southeastern winds [26] that influence drift, but most likely reflect historical stranding response effort. Much of the MS coast is accessible to the public and the Institute for Marine Mammal Studies (IMMS) began responding to dolphin strandings in 1984, so this area has historically had an unusually large response effort (140 of the Pre-Spill strandings analyzed in this study). The LA coast near Grand Isle is a relatively short coastline that is frequented by the public and the LA Department of Fish and Wildlife (LDWF), who reported 38 of the dolphin strandings considered in this study during the Pre-Spill period.

During the Active Response, clustering was only seen in LA from Grand Isle extending east into the MS River Delta and including Barataria Bay. The lack of clustering in other areas in the nGOM during the Active Response, specifically in areas that have had consistent stranding response (e.g., MS), may have resulted from an equal distribution of search effort and greater parity in reporting rates throughout much of the region. The exception was the spatial cluster in LA, which remained relatively unchanged after response-related records were removed, indicating that mortality was unique in this area relative to other areas within the nGOM. Analysis of the LA public/private and remote areas showed a 48% reduction of significant spatial clustering when response-related records were removed. Removal of response-related records restricted spatial clustering to areas in close proximity to Grand Isle and excluded remote portions of Barataria Bay potentially reflecting the pattern of spatial clustering that would have occurred in the absence of response efforts.

During the Post-RADC period, clustering was again seen along the MS mainland coast and highly accessible Ship Island and a second cluster was noted near Dauphin Island, AL. The Dauphin Island cluster may also be a function of increased response and reporting as the Alabama Marine Mammal Stranding Network (ALMMSN) at Dauphin Island Sea Lab (DISL) was established as a marine mammal stranding response facility during the Active Response phase of the oil spill (Ruth Carmichael, personal communication). Prior to the spill, DISL was not listed as a responder in Level A data used in this study, but played a large role in response during the Active Response phase (69 reported strandings considered in this study) and Post-RADC periods (48 reported strandings considered in this study). These results suggest that spatial clustering of strandings is representative of response effort across the nGOM, which should not be confused with actual mortality. Although estimating mortality relies on a better understanding of the influence of search and response efforts, how these results relate to actual mortality remains unknown.

The role of search effort in carcass detection has not been extensively studied, but increasing search effort (e.g., duration of search, number of searchers, spatial extent of search) would intuitively increase the number of carcasses found. A study conducted in Alaska to estimate avian carcass detection probability and persistence rates involved placement of carcasses on beaches and measuring detection rates when teams actively searched for carcasses. The authors determined that 40% of bird carcasses were found in one pass when people were actively searching and that a second pass resulted in a 70% detection rate [10]. While oil spill responders considered in this study were looking primarily for oil when surveying SCAT segments, responders were directed to report all injured or stranded animals that they encountered during surveys and clean-up activities. Also, dolphin carcasses are typically large (relative to birds) and thus would have high detection rates on beaches that were actively surveyed as part of the oil spill response. Thus, the influence of search effort on carcass recovery, while difficult to quantify, appears to have played an important role in carcass recovery during the Active Response period.

Carcass detection is difficult to link with mortality and often represents a small fraction of total mortality [7–9] as a variety of factors influence carcass detection [7–8,10–12]. A cetacean drift study conducted in France involved the release of 100 cetacean carcasses from a vessel 41 km from shore and noted that only 8% were later found stranded on beaches [9]. Williams et al. [13] estimated that cetacean carcass recovery rates for offshore species following the DWH oil spill was 2% (ranged from 0–6.2%, depending on species), suggesting that the total number of dead cetaceans could be 50 times the number that was recovered in the months immediately following the oil spill. Bottlenose dolphin recovery rates were not included because stock structure of the species was considered to be poorly understood in the nGOM [13,23]. In California, bottlenose dolphin stranding rates were estimated to represent 25% (95% CI: 0.20–0.33) of true mortality in a well-known inshore population; however, stranding records used to estimate mortality excluded those found on offshore islands as search effort in those areas was considered to be minimal [7]. Wells et al. [8] estimated bottlenose dolphin carcass recovery rates in a well-known population in Sarasota Bay to be 33% ± 0.170 SD. Sarasota Bay is believed to have high carcass recovery rates as conditions (e.g., wind, currents, etc.) often favor carcasses reaching shore quickly with fewer opportunities for scavenging and decomposition [8]. These studies show that carcass recovery rates vary spatially with environmental conditions and accessibility and they raise questions about the ability to understand true mortality rates of bottlenose dolphins in areas where the population is poorly understood, even with increased search effort and reporting.

The U.S. Department of Interior has finalized a Programmatic Damage Assessment and Restoration Plan and Programmatic Environmental Impact Statement that included estimates of reductions of near-shore dolphin populations resulting from the DWH oil spill. Estimates of oil-related mortality (difference in expected mortality from estimated actual mortality) of 22% (95% CI: 13–29%) were derived for the MS Sound and 35% (95% CI: 15–49%) for Barataria Bay, LA [27]. While these estimates are compelling, there is no mention of the effect of increased response activities on carcass recovery rates, from which estimates of mortality are derived. Coastal areas in the nGOM are a mixture of human-frequented and remote areas. Without estimates of Pre-Spill search effort and carcass detection rates, ascertaining the effects of changing search effort on mortality is difficult. It is impossible, based on current information, to determine what carcass detection rate is appropriate for the nGOM and more specifically for unique geographies within this region (e.g., barrier islands, salt marshes). Many of the dolphin stocks in the nGOM, as currently defined, overlap spatially and temporally [28–30], therefore a reported stranding is not necessarily from a particular population further confusing efforts to make robust inferences about population level impacts from stranding data. Updated population estimates are needed and greater effort should be expended to quantify carcass detection rates for known populations in this region. Long-term photo-identification studies designed to generate accurate estimates of bottlenose dolphin abundance including the use of mark-recapture models that can incorporate dead recovery [31], and greater understanding of spatial variation in search effort and carcass detection rates in the nGOM should help to generate meaningful estimates of mortality based on stranding data. Without this information, it will continue to be difficult to understand how strandings relate to overall mortality and population trends in this region.

### Limitations

There were several limitations in this study that should be considered. First, stranding records with erroneous coordinates were removed prior to analysis because accurate spatial location of strandings was critical for several facets of the analysis. Improvement and access to GPS technology increased during the study period and likely improved the accuracy and reporting rates of GPS coordinates, resulting in fewer erroneous and missing coordinates in Level A data across the temporal extent of the study. Also, stranding records within 161 m of an oil spill response team were identified as being response-related because it was assumed that strandings occurring within this distance on the day a segment was surveyed would likely have been detected by response teams. Ideally, Level A data would include more information on the reporting party; however, this information was rarely included. As such, it must be acknowledged that some records classified as response-related may have been reported by parties unrelated to the response effort or could have been reported by the public in the absence of response personnel. However, strandings that occurred in remote areas were most likely reported by response teams as these areas are infrequently visited by other potential observers. Also, most bottlenose dolphins die at sea and drift for a period before making landfall. Oil spill responders were working SCAT segments along shorelines impacted by floating oil, which also drifted with ocean currents before coming ashore. Thus, there was considerable overlap in cetacean carcass and oil coverage throughout the AOR, which likely increased the probability of carcass detection by response teams. Further, the 161 meter buffer from the designated SCAT segments may be too conservative as some strandings located further than 161 m from a SCAT segment may have also been reported by response teams while on-site or traveling to and from the SCAT segments. These strandings would not have been classified as response-related unless specifically noted in the Level A comments. Also, there was no attempt to quantify other factors that would increase reporting of strandings after the oil spill. These include increased awareness and media coverage of dolphin strandings among municipal, state, and federal agencies, conservation organizations, and the general public, increased response among the stranding network (particularly in remote regions of LA and AL), and increased availability of cellular and smart phone technologies. Thus, the public became increasingly more aware of strandings and had increased capacity to obtain location information and to photograph strandings (which is all that is needed for reported strandings to receive a Level A report with species identification). Because the DWH oil spill was a manmade disaster that lasted for a prolonged period, coastal residents may have been more willing to report cetacean carcasses as there was considerable outreach to document the results of the oil spill [27]. These factors must be considered in future studies of carcass detection and in determining its relation to mortality rates.

### Conclusions

The results presented here provide evidence to suggest that increased search effort during the DWH oil spill, specifically search effort associated with the DWH response, increased the frequency and the spatial and temporal extent of dolphin stranding reports in the nGOM. These findings do not preclude that the total number of strandings (both reported and unreported) actually increased during this time. Instead these results show that search effort resulting from the DWH oil spill response, including those in remote portions of the UME geographic region, had the capacity to increase reporting of dolphin strandings, regardless of the actual mortality rate. Undoubtedly, more work needs to be done to quantify population size and trends within the nGOM and to quantify carcass detection rates including the role of search effort to more completely understand how stranding data relates to actual mortality. This is vital for understanding the status and trend of a protected species within the nGOM.

## Supporting information

S1 Table Level A bottlenose dolphin (*Tursiops truncatus*) stranding data for the study period (1996–2015) within selected areas of the northern Gulf of Mexico. (XLSX)

S2 Table Bottlenose dolphin (*Tursiops truncatus*) strandings cross-referenced with oil spill response activities within selected areas of the northern Gulf of Mexico. (XLSX)
